# Case Report: ETV6 c.744del gene mutation acute lymphoblastic leukemia in identical twins

**DOI:** 10.3389/fped.2026.1808862

**Published:** 2026-06-15

**Authors:** Xiaoxiao Xu, Huiyan Yang, Ruihan Fang, Lin Tan, Yaxin Luo, Fuyu Pei, Xuedong Wu

**Affiliations:** 1 Center of Pediatrics, Nanfang Hospital, Southern Medical University, Guangzhou, China; 2 Department of Pediatric Hematology, Shenzhen Children's Hospital, Shenzhen, China

**Keywords:** acute lymphoblastic leukemia, children, ETV6 gene, germline mutation, twins

## Abstract

**Background:**

It is extremely rare for monozygotic twins to both develop leukemia. This paper reports a clinical case in which a pair of monozygotic twins with germline ETV6 gene mutations successively developed acute lymphoblastic leukemia (B-cell type, ALL-B). The ETV6:c.744del (p.S248fs) is a novel germline mutation associated with familial susceptibility to hematologic malignancies. This mutation site has not been detected in relevant databases or literature, and this is the first international report of it. It provides reference value for genetic screening of such diseases.

**Case introduction:**

The elder twin (2 years old) presented with fever and skin petechiae; the younger twin (4 years old) presented with knee joint pain and lymphadenopathy. Both were diagnosed with B-cell ALL. Germline ETV6 c.744del was confirmed using DNA from buccal swabs and DNA-based next-generation sequencing. The mother and maternal uncle are carriers; the father is wild-type. The younger twin carried additional somatic NF1 and KRAS mutations.

**Outcomes:**

The elder twin achieved complete remission and has remained disease-free for 42 months. The younger twin relapsed during maintenance therapy and underwent allogeneic hematopoietic stem cell transplantation from their uncle (non-carrier,their father's younger brother), achieving remission.

**Conclusion:**

The successive occurrence of ALL-B with germline ETV6:c.744del mutation in the twins suggests genetic susceptibility to the disease. The frameshift mutation of ETV6:c.744del may serve as a potential driving factor. It is recommended that germline genetic testing and family screening be conducted for all pediatric patients with familial clustering of leukemia.

## Introduction

Acute lymphoblastic leukemia (ALL) is the most prevalent hematologic malignancy in children and a leading cause of morbidity and mortality among pediatric populations (1–3). Currently, the genetic factors predisposing individuals to ALL remain poorly understood. Traditionally,genetic susceptibility has not been considered a primary risk factor for ALL development; however, a growing body of evidence suggests that its role far exceeds previous perceptions. For instance, in familial ALL (4), although the genetic basis of many familial cases remains unclear, studies indicate that children with affected siblings face a 2 to 4 fold increased risk of developing ALL, underscoring the significance of genetic predisposition (5). Recently,germline gene mutations, such as those in ETV6 (6, 7), have been identified in both familial and sporadic cases of ALL. This report describes a rare case of monozygotic twins who sequentially developed ALL, while also revealing that the family carries a rare pathogenic ETV6 variant.

## Methods

### Germline mutation confirmation

To confirm the germline origin of the ETV6 c.744del mutation, DNA was extracted from oral epithelial cells (non-hematopoietic tissue) from both twins and from peripheral blood of family members. Sanger sequencing and DNA-based next-generation sequencing (NGS) were performed.

### RNA sequencing and fusion gene analysis

Whole transcriptome sequencing (RNA-seq) was performed on diagnostic bone marrow samples from both twins using standard protocols. A targeted panel of 46 common ALL-associated fusion genes was also tested.

### mRNA expression analysis

Total RNA was extracted from fresh peripheral blood using Trizol reagent (Invitrogen, USA). Reverse transcription was performed with HiScript II Q RT SuperMix (Vazyme, China). Quantitative real-time PCR (qPCR) was conducted using ChamQ SYBR qPCR Master Mix (Vazyme, China) on a CFX96 system (Bio-Rad). The 2^−*ΔΔ*CT^ method was used. Primers for ETV6: forward 5’-TCGACGCCACTTCATGTTCC-3’, reverse 5’-TTTTCAGCCCACTTGAGCCA-3’; for GAPDH: forward 5’-ATCAGCAATGCCTCCTGCAC-3’, reverse 5’-TGGCATGGACTGTGGTCATG-3’. The mutant group included both twins and their mother (carriers); the wild-type group included the father and two healthy children (the same age selected at random).

### Three-dimensional structural analysis

Amino acid sequences of wild-type and mutant ETV6 proteins were obtained using Snapgene. Three-dimensional structures were predicted using I-TASSER (https://zhanggroup.org/I-TASSER/).

## Case description

### Elder twin

A 2-year-old male presented to our hospital in July 2022 with fever and scattered skin petechiae. Physical examination revealed the liver palpable 4 cm below the right costal margin(soft), no splenomegaly, and several enlarged lymph nodes in the neck and inguinal regions (soft, clear borders, up to 1.5 cm × 1.5 cm). There was no notable family history of hematologic malignancies or bleeding disorders. Laboratory tests showed white blood cell count 130.36 × 10^9^/L, total lymphocytes 113.67 × 10^9^/L, hemoglobin 56 g/L, and platelets 9 × 10^9^/L. Bone marrow morphology revealed 94% primitive/immature lymphocytes. Flow cytometry identified primitive/immature B lymphocytes accounting for 89.3% of total nucleated cells, with immunophenotype: CD19+, CD10+, CD34-, HLA-DR+, partially CD9+, CD33-, CD117-, CD3-, CD56-, CD2-, partially CD20+, CD22+, sIgM-, CD5-, CD66c-, CD36-, CD61-, CD38+, CD7-, CD123-, CD11b-, CD13-, CD15-, CD14-, CD64-, partially cCD79a+, cCD22+, cu-, partially nTDT+, cCD3-, cMPO-. RNA-seq detected the ETV6 S248fs mutation, as well as three distinct fusion events: an ERG-chr12 rearrangement, a PAX5-ZCCHC7 rearrangement, and a chr12-ERG fusion gene. A targeted panel of 46 common ALL-associated fusion genes was negative. (Note: The detected fusion genes were uncommon variants not included in the standard 46-fusion panel; thus, no discordance exists). The patient was diagnosed with B-cell ALL. He received chemotherapy per the CCCG-ALL 2020 intermediate-risk protocol. Complete remission (bone marrow MRD < 0.01%) was achieved on both day 19 and day 46 of induction therapy. The patient has remained in continuous remission for 42 months of follow-up.

### Younger twin

A 4-year-old male, the identical twin of the above patient, presented on February 2, 2024, with left knee joint pain and lymphadenopathy. Physical examination showed no hepatosplenomegaly; several soybean-sized, mobile lymph nodes were palpable in the neck and inguinal regions. There was no prior history of thrombocytopenia or bleeding. Laboratory tests: white blood cell count 43.81 × 10^9^/L, total lymphocytes 32.60 × 10^9^/L, hemoglobin 98 g/L, platelets 48 × 10^9^/L. Bone marrow morphology showed 93.6% primitive/immature lymphocytes. Flow cytometry identified primitive/immature B lymphocytes accounting for 80.5% of total nucleated cells, with immunophenotype: CD19+, CD10+, partially CD34+, HLA-DR+, CD9+, CD33-, CD117-, CD3-, partially CD56+, CD2-, CD20-, CD22+, sIgM-, CD5-, CD66c-, CD36-, CD61-, dim CD38+, CD7-, partially CD123+, CD11b-, CD13-, CD15-, CD14-, CD64-, cCD79+, cCD22+, cu-, partially nTDT+, cCD3-, cMPO-. RNA-seq detected the same ETV6 S248fs mutation, plus somatic NF1 E1919* and KRAS K147N mutations, and the SENP7::TTC28 fusion gene. A targeted panel of 46 common ALL-associated fusion genes also negative. He was diagnosed with B-cell ALL and treated with the CCCG-ALL 2020 low-risk protocol. On day 19 of induction, bone marrow MRD was 0.28%; complete remission (MRD < 0.01%) was achieved by day 46. However, during early maintenance therapy (more than 10 months after diagnosis), bone marrow MRD rose to 1.44% with 15% primitive/immature lymphocytes morphologically,indicating relapse. He then underwent allogeneic hematopoietic stem cell transplantation from their uncle (their father's younger brother) who did not carry the ETV6 mutation. Donor selection avoided related carriers (mother and maternal uncle) to prevent re-transmission of the germline predisposition. The patient is currently in remission.

### Genetic testing

**Figure 1:**
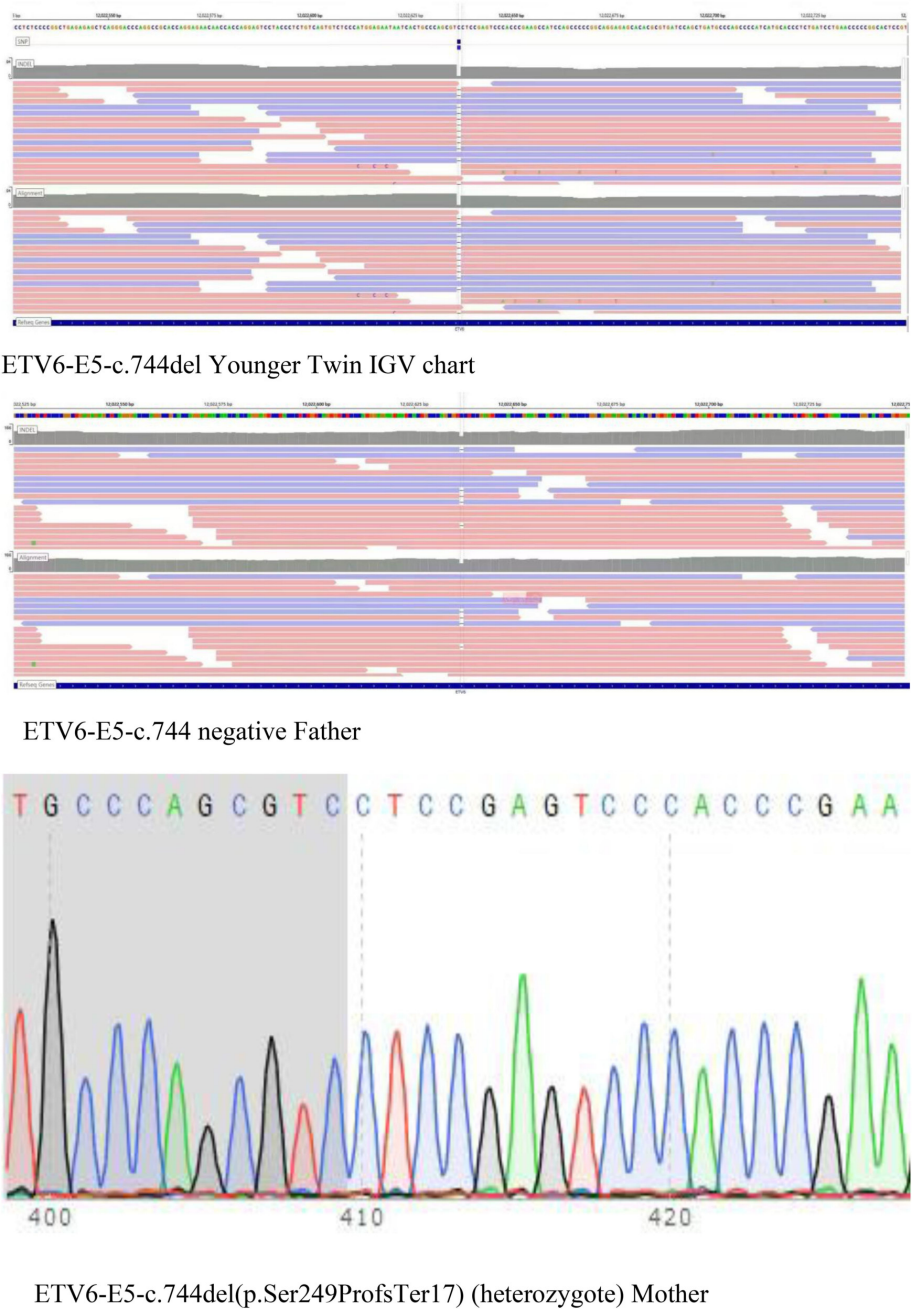

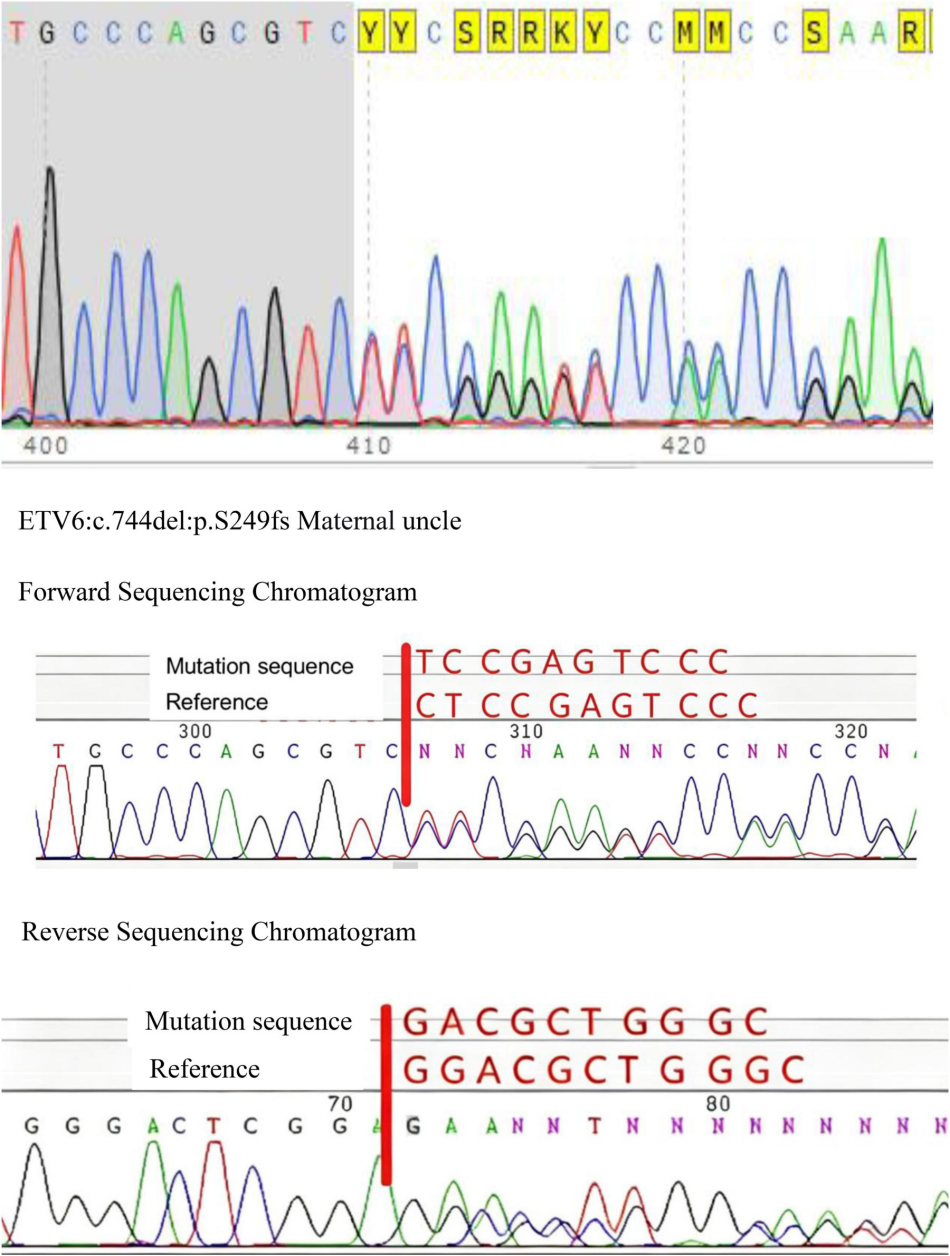
Sanger sequencing confirming the heterozygous ETV6:c.744del:p.S249fs variant analyzed by mutation pedigrees.

**Figure 2:**
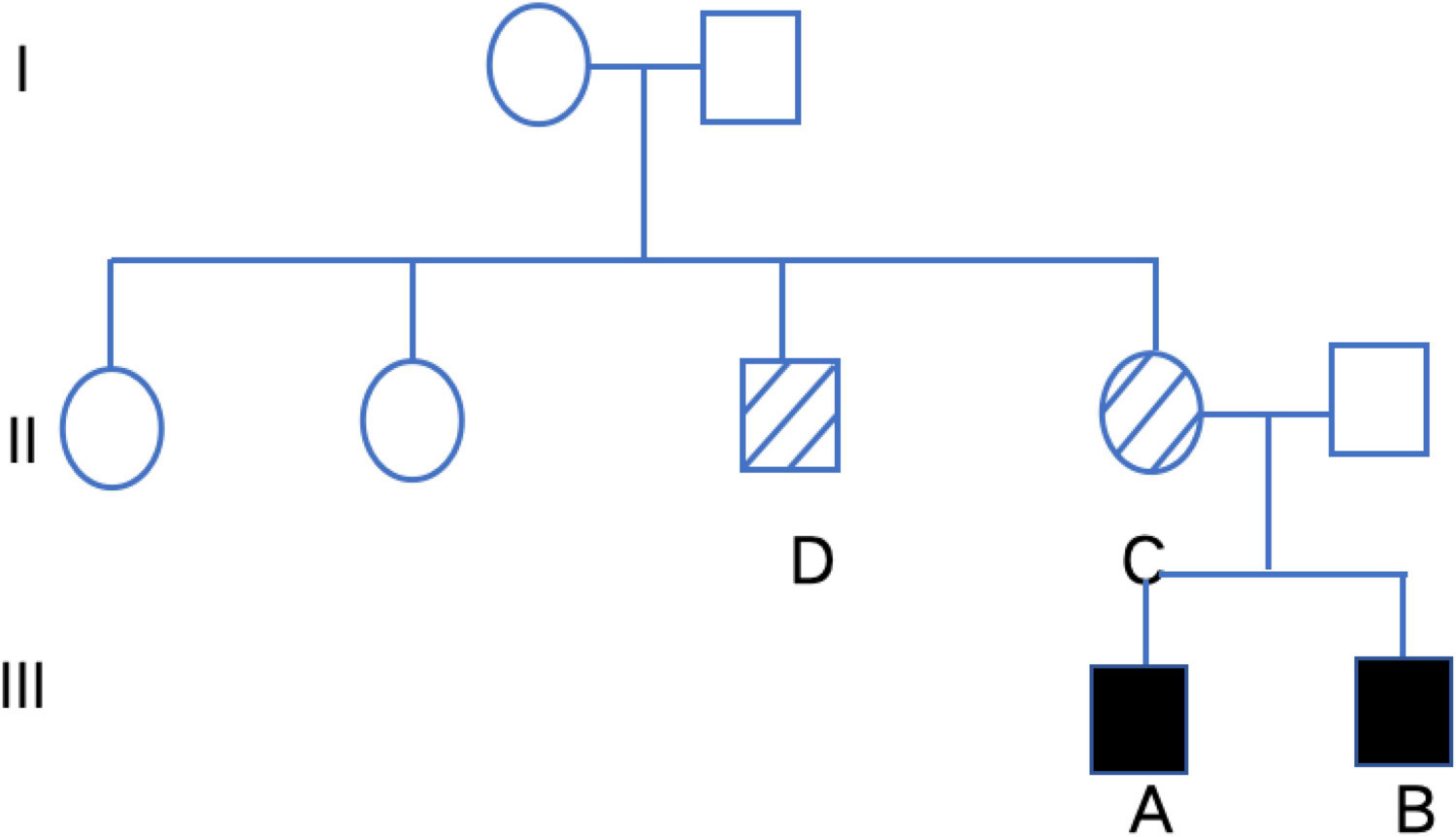
Pedigree showing the segregation of the ETV6:c.744del:p.S249fs variant within the extended family of the proband (patient A, B). C and D are all carriers. Individual D (maternal uncle) and D ’s children are in good health so far, but children not undergone genetic testing.

The germline ETV6:c.744del (p.S248fs) frameshift mutation was identified in both twins. PCR Sanger sequencing confirmed that the mother and maternal uncle are heterozygous carriers, while the father is wild-type. To avoid confusion, we have added IGV charts from the twins as an additional panel to clearly demonstrate the heterozygous mutation (shown below as a representative image) in Figure 1. The family pedigree is shown in Figure 2.

### Expression analysis of ETV6 mRNA

Peripheral blood samples from the two patients, their parents, and two unrelated healthy children (same age, selected at random) were analyzed. ETV6 mRNA expression levels were measured by qPCR using the 2^−*ΔΔ*CT^ method. The twin patients and their mother comprised the mutant (MUT) group; the father and the two healthy children comprised the wild-type (WT) control group.

### RNA-Seq allele-specific expression analysis

**Figure 3:**
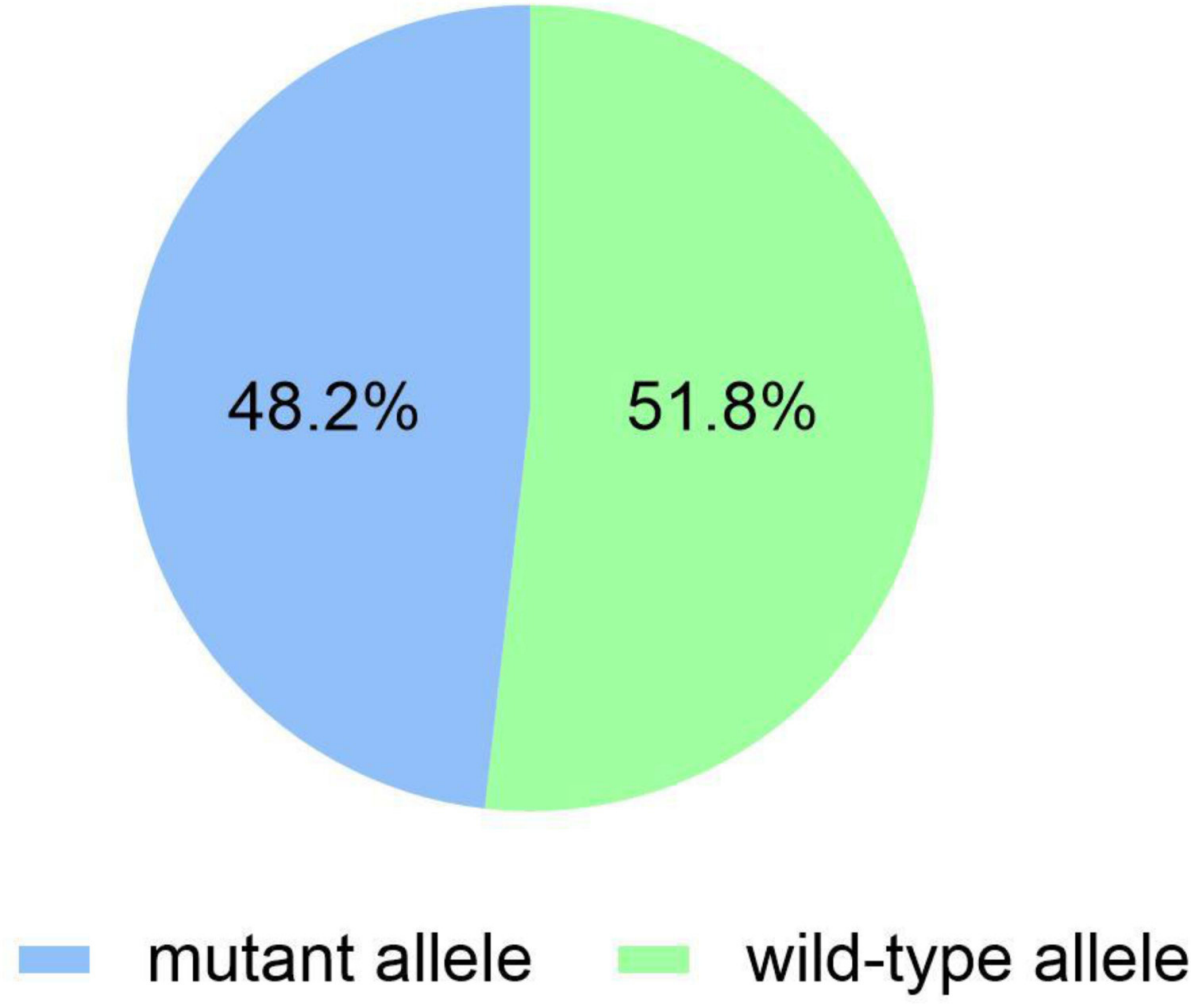
The proportion of mutant and wild-type allele.

To assess the expression levels of the two ETV6 alleles, we analyzed the RNA-seq data from diagnostic bone marrow samples of both twins. The results showed that both alleles were expressed at similar levels in both patients (mutant allele: 48.2% of total ETV6 reads; wild-type allele: 51.8%). The data is presented in a pie chart as Figure 3. This indicates that the ETV6:c.744del frameshift does not significantly affect transcript stability or trigger nonsense-mediated decay.

### Three-dimensional structural analysis of wild-type and mutant protein

The amino acid sequences of wild-type and mutant proteins were obtained through Snapgene software. We obtained the predicted Three-dimensional (3D) structural of wild-type and mutant proteins by uploading the amino acid sequences to Iterative Threading ASSEmbly Refinement (I-TASSER). The prediction can be found at the website (https://zhanggroup.org//I-TASSER/).

### ETV6 mRNA expression

**Figure 4:**
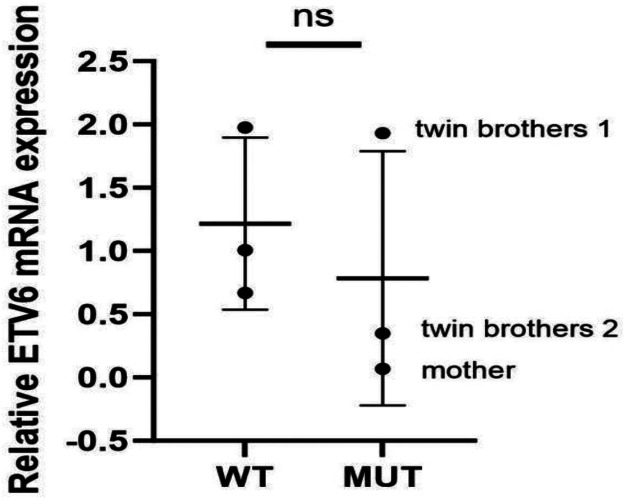
The relative ETV6 mRNA expression in WT group and MUT group. Each symbol represents an individual qPCR replicate (triplicate measurements). Bars show mean ± SD. For the mutant samples, the higher dot is the one with active disease while the two lower dots are not.

The peripheral blood samples were taken from the older brother was in remission, while the younger brother was in the active phase of the disease. qPCR was performed in triplicate for each sample. Figure 3 has been replotted as a scatter plot showing individual data points (each symbol represents one replicate) overlaid on bars representing mean ± SD. The variability reflects both biological replicates and technical artifacts. No significant difference in ETV6 mRNA expression was observed between the MUT group (0.7836 ± 1.004) and the WT group (1.217 ± 0.6794) (*p* = 0.5696, unpaired t-test) (Figure 4).

### 3D structure of wild-type and mutant ETV6 proteins

**Figure 5:**
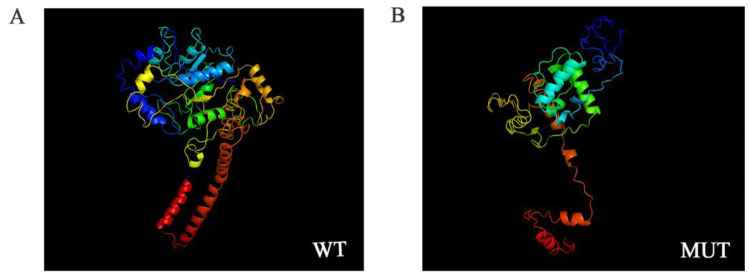
The 3D structure of wild-type **(A)** and mutant **(B)** ETV6 proteins.

The I-TASSER prediction revealed significant alterations in the mutated protein's 3D structure (Figure 5). The deletion causes a frameshift mutation that generates a stop codon TGA, triggering premature termination of translation and producing a truncated protein. The truncated protein lacks part of the *α*-helix and *β*-sheet structures, which may affect the biological function of the protein.

## Discussion

The ETV6 gene (also known as TEL) is located on chromosome 12p13 and is a member of the ETS family of transcription factors. In 2015, germline mutations in the ETV6 gene were identified in families with thrombocytopenia and hematologic malignancies (6, 8). It has been reported that approximately 30% of patients with hematologic neoplasms carry a mutation in this gene, including ALL-B (the most common), MDS, AML, and mixed-phenotype acute leukemia, among others. The incidence of germline *ETV6* mutations remains unclear, and its primary function is to encode a transcription regulator involved in hematopoiesis and platelet development. In the present family, neither the twins nor the carrier mother or maternal uncle had documented thrombocytopenia or abnormal bleeding histories, highlighting incomplete penetrance and variable expressivity a common feature of ETV6 germline disorders. Germline gene mutations typically affect the ETS DNA-binding domain, leading to impaired nuclear localization and, consequently, reduced transcriptional repression by ETV6, as well as decreased synthesis of platelet precursors (9). This domain is part of the leukemia fusion proteins, suggesting that alterations in the expression of normal ETV6 target genes are involved in the pathogenesis of leukemia (10). However, conventional analytical methods (such as immunofluorescence and FISH) may fail to identify such structural variants, necessitating the use of comprehensive whole transcriptome sequencing (RNA-seq) and/or whole genome sequencing, highlighting the importance of gene structural analysis in assessing genetic susceptibility to leukemia (11).

A notable characteristic of familial germline ETV6 mutations is the inherited susceptibility to hematologic malignancies, with ALL being the most prevalent (12). ETV6 is highly expressed in early hematopoietic progenitor cells and plays a crucial role in bone marrow hematopoiesis. The protein consists of three functional domains: the N-terminal conserved pointed (PNT) domain mediates homodimerization and heterodimerization, while the C-terminal ETS domain is essential for DNA binding to core sequences recognized by other *ETS* family members.These two domains are connected by a central linker domain, which plays a role in nuclear export, co-factor binding, and transcriptional repression (13). Germline *ETV6* variants are present *in utero*, indicating that leukemogenesis occurs at an early stage. Unlike somatic mutations in ETV6, germline variants increase the risk of developing ALL by 1.5 to 2 fold (13). Missense variants retain dimerization with wild-type ETV6 and exert a potential dominant-negative effect, revealing the profound impact of germline *ETV6* mutations on leukemia transcription factors. This mutation results in a truncated protein lacking the ETS domain, which is predicted to impair nuclear localization. Not all germline mutations progress to leukemia; additional somatic mutations (e.g., KRAS, NRAS, NF1) are required (14). This is exemplified by the twin patients in this case, who, despite sharing the same *ETV6* mutation and living environment within the same family, developed ALL sequentially. Whole transcriptome sequencing revealed that the younger twin carried somatic gene mutations in *NF1* and *KRAS*, which are clearly associated with ALL, potentially contributing to his later onset and early relapse in a synergistic manner. Further research is needed to elucidate the specific mechanisms involved.

The mRNA expression analysis showed no significant difference between mutant and wild-type groups, suggesting that the c.744del frameshift does not trigger nonsense-mediated decay or alter transcript stability. Thus, the pathogenic mechanism likely operates at the protein level. The 3D structural prediction indicates a truncated protein lacking the ETS domain. This domain is essential for nuclear localization and DNA binding; its loss is predicted to mislocalize ETV6 to the cytoplasm, abrogating its transcriptional repressor function. Indeed, prior studies have shown that germline ETV6 mutations often disrupt nuclear localization, which is central to leukemogenesis (9, 13). The novel mutation described here is predicted to similarly impair ETV6 nuclear localization, although direct immunofluorescence studies would be required to confirm this.

Although both the patient's mother and maternal uncle carry the same ETV6 variant, they have not developed any form of tumor to date, underscoring incomplete penetrance. Given the rarity of this genetic predisposition, both the incidence rate and the associated cancer risk remain unpredictable. Currently, there is no established appropriate monitoring method for individuals carrying pathogenic ETV6 variants. For now, the key priorities are to provide genetic counseling and regular follow-up. For the younger twin's allogeneic transplantation, their uncle (non-carrier, their father's younger brother) was selected to avoid re-transmitting the germline mutation a critical consideration when transplanting patients with known germline predisposition syndromes. Related donors should be screened for the familial mutation, and carrier donors generally avoided unless no alternative exists. When necessary, multidisciplinary clinical and laboratory teams should be engaged to conduct family studies, although the specific data obtained regarding the variant may sometimes be limited.

Germline and somatic ETV6 mutations are frequently detected in cancer patients; however, more data are needed to distinguish disease-associated alleles from harmless polymorphic mutations. The spectrum of malignancies or other diseases associated with specific ETV6 mutations remains unclear. Importantly, there is an urgent need to better understand the physiological functions of ETV6 and to further elucidate the molecular pathology of its mutants. This could facilitate risk stratification and medical management for affected individuals and potentially lead to novel targeted interventions for treating malignancies. In cases of unexplained familial hematologic malignancies, germline gene validation combined with structural variant analysis should be performed. The identification of germline susceptibility provides a reference for the diagnosis and follow-up of high-risk relatives and other at-risk individuals, and its underlying mechanisms warrant further investigation.

## Data Availability

The original contributions presented in the study are publicly available. This data can be found here: https://www.ncbi.nlm.nih.gov/bioproject/PRJNA1475771, Accession number: PRJNA1475771.
